# Parental differential treatment and symptoms of child psychopathology: A twin study

**DOI:** 10.1017/S0954579426101370

**Published:** 2026-05-05

**Authors:** Janna Pickett Maravilla, Courtney Lyding, Sierra Clifford, Harold Hill Goldsmith, Kathryn Lemery-Chalfant

**Affiliations:** 1 Psychology, Arizona State University, USA; 2 Psychology, University of Wisconsin-Madison, USA

**Keywords:** parental differential treatment, psychopathology, twin study, sibling relationships, parent–child relationships

## Abstract

Parental differential treatment is associated with higher levels of psychopathology symptoms in children. Both higher overall levels of differential treatment (absolute/magnitude of differential treatment) and consistently favoring one child over another (relative differential treatment) are associated with risk in children. This study enhances understanding of parental differential treatment using a genetically informed twin design that clarifies child- and parent-driven effects. Participants included 632 twin pairs (*M*age = 7.6 years, SD = 0.94; 96% White, 44% Rural) and parents. Parental differential treatment was assessed using an observed card game interaction and reports from mothers, fathers, and children. Twin modeling indicated heritable influences on parental hostility (*h* ^2^ = .34 for females, .06 for males) and intrusiveness (*h* ^2^ = .51 across the sample), suggesting that children’s heritable traits elicit parenting. Observed intrusiveness differences predicted ADHD. Absolute and relative differences in maternal discipline predicted externalizing, internalizing, and ADHD symptoms, with a similar but less strong pattern for paternal discipline. However, absolute differences in paternal affection and paternal partiality proved especially important for children’s psychopathology. Findings show children’s behavior can elicit maladaptive differences in parenting, informing interventions.

## Parental differential treatment and symptoms of child psychopathology

One in six U.S. youth (aged 6–17) (Whitney & Peterson, 2019) and one in five U.S. adults (Substance Abuse and Mental Health Services Administration, 2021) have a diagnosed mental health disorder. For an estimated fifty percent of the individuals facing lifelong struggles with these disorders, the onset of symptoms occurs before age 14 (Kessler, 2005). Thus, identifying risk factors for child psychopathology assumes crucial importance. Classic findings in the behavioral genetics literature indicate that non-shared environmental factors (i.e., factors that make children in the same family less alike) influence most youth outcomes to a greater degree than shared environmental factors (Plomin & Daniels, 1987; Turkheimer & Waldron, 2000). Parental differential treatment, a broad term for any parenting behavior that differs between siblings, is one non-shared environmental influence that is highly salient for children and may contribute to the development of psychopathology (Jensen & Thomsen, 2024). At the same time, genetic factors can drive both child behavior and the parenting that the child receives, and a portion of the association between parental differential treatment and child psychopathology probably also results from the child’s own heritable behavioral patterns (Loehlin et al., 2005). As a result, understanding both genetic and environmental influences on parenting is necessary for a comprehensive understanding of parental differential treatment as a risk factor for child psychopathology.

The focus of the study was two-fold: (1) to examine the origins of parenting through understanding child-driven heritable contributions to the parenting that they receive and (2) to examine the association between parental differential treatment and psychopathology.

### Heritability of parenting

Although parenting is often considered a purely environmental factor, parents may treat their children differently in response to differences between their children (Bell, 1968), suggesting the possibility that parental differential treatment might be a response to, rather than the cause of, psychopathology symptoms. Bronfenbrenner and Ceci’s bioecological model (1994) emphasizes children‘s role in actively engaging with and eliciting behavior from parents, which may be driven by heritable characteristics, and genetically informed research supports the importance of child influences on parenting (Avinun & Knafo, 2014).

Behavior genetic researchers use sibling and twin studies to separate the variance in measured variables into genetic and environmental components, relying on information about degrees of genetic kinship and environmental similarity assumed to reflect common experience (Lemery & Goldsmith, 1999). Because monozygotic (MZ) twins share 100% of their genetics, dizygotic (DZ) twins and full siblings share an average of 50%, and adoptive/step-siblings share 0%, differing degrees of trait similarity among these kinship groups can be used to estimate how much of the variance in that trait is associated with genetic or environmental sources. When siblings who share more genomics are more similar in their behavior than siblings who share less genes, genetic influences are inferred to play a role, provided that the assumption of equal environmental similarity across kinship groups holds. When behavioral similarity is not aligned with genetic similarity (e.g., when MZ and DZ twins share the same degree of behavioral similarity), the inference is that individual differences in the behavior are not genetically rooted. Kinship studies implicate both genetic and environmental sources of variation in parenting and differential treatment.

A meta-analysis by Avinun and Knafo (2014) found that parenting (combined across maternal and paternal negativity and positivity) was 23% heritable (95% *CI* = [20, 25]), suggesting that children’s own heritable traits may influence parenting. Heritability estimates did not differ significantly across maternal and paternal parenting or positive and negative parenting. These genetic influences on parenting observed in child-based twin studies reflect evocative gene–environment correlation (evocative rGE) (Plomin et al., 1977), in which children’s genetically influenced characteristics elicit responses from parents. Parental differential treatment also seems to be influenced by children’s heritable traits (Carbonneau et al., 2002b; Daniels & Plomin, 1985; Pike et al., 2000), but the magnitude of heritability estimates may differ depending on scale and whether the parent or child is reporting the treatment. Carbonneau et al. (2002b) found significant genetic influences on all four of the Twin Inventory of Relationships and Experiences (TIRE) scales from mother report, father report, and twin report, except for mother’s reports of their own preferences in female twin pairs, which showed low heritability (13%). This pattern suggests that reporter and/or sex differences may play a role in the heritability of differential treatment. One examination of the SIDE, which measures differential affection and differential control rather than parental criticism, dominance, and preference, detected genetic effects on child reports of differential maternal and paternal control and affection (Pike et al., 2000). However, another examination of the SIDE, which compared adopted siblings to non-adopted siblings, found no genetic effects (Daniels & Plomin, 1985).

Although the existing literature supports heritable, child-driven effects on parenting and differential treatment, current research is limited and scattered based on age and measurement type. Very few studies have used behavior genetic methods to examine parental differential treatment, because twin analyses typically require separate scores for each twin. This requires measuring each twin’s perception of parental differential treatment, having parents rate their behavior toward each twin separately, or conducting separate observations of parenting behaviors for each twin and then drawing conclusions about differential treatment. More research is needed to thoroughly examine how children’s heritable traits may influence differential parenting which is addressed in the present study.

### Associations between parental differential treatment and child psychopathology

Although parental differential treatment may partly reflect child-driven genetic influences, it likely also remains a powerful non-shared environmental influence with implications for children’s risk for psychopathology. Examining these associations necessitates careful consideration of how differential treatment is defined and measured.

Inspired by social comparison theory (Festinger, 1954) which suggests that children who are favored by differential treatment have better mental health compared to children who are disfavored, and Adler’s theory of individual psychology (Ansbacher & Ansbacher, 1956) which suggest that children in families with less overall parental differential treatment have better mental health, parental differential treatment (PDT) is typically examined using both relative and absolute measures. Relative measures capture the direction of favoritism, identifying which sibling receives more favorable treatment, and studies taking a relative approach focus on differences between favored and disfavored children (Eradus et al., 2024; Meunier et al., 2012; Richmond & Stocker, 2008; Shanahan et al., 2008). With relative measures, such as the Sibling Inventory of Differential Experience (SIDE; Daniels & Plomin, 1985) or the Sibling Relationship Questionnaire (SRQ; Furman & Buhrmester, 1985), participants are asked to indicate who is favored (Luo et al., 2020; Richmond et al., 2005), or researchers calculate difference scores from sibling ratings (McHale et al., 2000). Absolute measures quantify the magnitude of differential treatment regardless of direction, either by asking siblings to report overall differences in treatment (e.g., “To what extent are you treated differently from your sibling?”; Kowal et al., 2002) or via researchers computing the total discrepancy between ratings (Atzaba-Poria & Pike, 2008; Boyle et al., 2004). These measures assess general inequality rather than direction and are often used to examine the overall presence of PDT (Buist et al., 2013; Jenkins et al., 2003).

A growing body of research demonstrates that both relative (Carbonneau et al., 2002a; Eradus et al., 2024; Luo et al., 2020; Meunier et al., 2012; Richmond & Stocker, 2008) and absolute (Atzaba-Poria & Pike, 2008; Boyle et al., 2004; Buist et al., 2013; Jenkins et al., 2003; Kowal et al., 2002) measures of parental differential treatment are associated with children’s psychopathology symptoms (Jensen & Thomsen, 2024). Across studies, PDT is more consistently linked to externalizing than internalizing symptoms (Boyle et al., 2004; Buist et al., 2013; Eradus et al., 2024; Jeannin & Van Leeuwen, 2015; Jensen & Thomsen, 2024; Kowal et al., 2002; Luo et al., 2020; Meunier et al., 2012; Richmond et al., 2005; Richmond & Stocker, 2008), likely because externalizing behaviors represent more visible and reactive responses to differential discipline, especially in parent-reported data. Children’s externalizing behaviors might also elicit more differences in parenting. Internalizing symptoms tend to relate more strongly to perceptions of unfairness in PDT (Kowal et al., 2002; McHale et al., 2000) and are more pronounced in adolescence, when such emotional experiences peak (Buist et al., 2013; Papachristou & Flouri, 2020). Although ADHD is sometimes grouped with externalizing disorders, its etiology is distinct: ADHD is highly heritable (Rietveld et al., 2003), whereas externalizing problems show moderate heritability (Towers et al., 2000). Given its behavioral overlap with externalizing symptoms, differential discipline may also predict ADHD-related behaviors (Schachar & Tannock, 1995).

PDT can also occur across several domains of parenting, including warmth (Atzaba-Poria & Pike, 2008; Jenkins et al., 2003; McHale et al., 2000; Shanahan et al., 2008), positive interactions (Boyle et al., 2004; Jeannin et al., 2015; Kowal et al., 2002; Meunier et al., 2012), hostility (Atzaba-Poria & Pike, 2008; Boyle et al., 2004; Richmond & Stocker, 2008), punishment (Jenkins et al., 2003; Richmond et al., 2005), chore allocation (McHale et al., 2000), and control (Jeannin et al., 2015; Kowal et al., 2002). Across studies, differences in negative parenting, such as hostility, criticism, or punitive control, are the most robust predictors of internalizing and externalizing symptoms (Boyle et al., 2004; Eradus et al., 2024; Jensen & Thomsen, 2024; Meunier et al., 2012; Richmond & Stocker, 2008; Shanahan et al., 2008). In contrast, differences in warmth are linked to fewer symptoms (Atzaba-Poria & Pike, 2008; Jenkins et al., 2003; McHale et al., 2000). These effects likely arise because disparities in negative parenting (e.g., one child being scolded while another is not) are more salient and emotionally impactful than subtler differences in positive interactions. Some studies assess PDT as overall favoritism rather than domain-specific behavior (Jeannin et al., 2015; Luo et al., 2020; McHale et al., 2000). Absolute favoritism generally predicts higher levels of externalizing (Jeannin & Van Leeuwen, 2015; Luo et al., 2020) and internalizing symptoms (Luo et al., 2020), though not all findings are consistent (Meunier et al., 2012).

Existing research on PDT is limited by a narrow developmental focus and inconsistent methodology. A recent meta-analysis highlighted the need for studies to examine child-driven effects and to incorporate multiple operationalizations of PDT to clarify how measurement affects links to psychopathology (Jensen & Thomsen, 2024). Theories of development further suggest that PDT may be especially relevant during middle childhood (ages 6–12), when children are highly sensitive to parental behavior yet still spend substantial time with parents (Bronfenbrenner & Ceci, 1994; Buist et al., 2013; Steinberg & Monahan, 2007). According to Piaget’s (1954) cognitive theory of development, children in this period are aware of differential treatment but may interpret it in concrete terms, lacking the abstract reasoning needed to appreciate nuances in parenting behavior. These developmental characteristics make middle childhood a critical and understudied window for understanding how PDT relates to psychopathology. To address these gaps, the present study used a twin design to examine genetic and environmental influences on parental differential treatment. In addition, we incorporated both relative and absolute measures, included reports from mothers, fathers, and children, and utilized observational data across multiple parenting domains during the salient developmental period of middle childhood.

### The current study

Our first goal was to examine the genetic and environmental influences on observed parenting using the twin method. Because these models require an independent score for each twin to estimate the genetic and environmental influences, we focused on the observational assessments. We expected that parenting would be partially explained by genetics and that there would be shared genetic influence on psychopathology symptoms and parenting through evocative rGE.

A second goal was to relate reports of parental differential treatment to child symptoms of internalizing, externalizing, and ADHD. Because past literature shows strong ties between differential negative parenting and externalizing symptoms, we expected that both absolute and relative parental differential treatment in hostility, intrusiveness, and discipline would predict externalizing symptoms and ADHD. We also predicted that differential hostility, intrusiveness, and discipline would be related to internalizing behaviors, but as this association is not as well established in the literature, we predicted that the association would be weaker compared to externalizing symptoms. No formal expectations were stated for differential attention and affection, as the literature for these aspects of differential parenting is inconclusive. We also hypothesized that measures of absolute maternal and paternal partiality (i.e., the overall amount of favoritism in a family) would predict higher internalizing, externalizing, and ADHD symptoms.

Finally, to add to the literature on measurement comparisons, we examined multiple measures of parental differential treatment, including reports from mothers, fathers, and children, as well as observational assessments in the home environment. We anticipated strong agreement among different reported measures of differential treatment, but only weak agreement between reported and observed measures (Brody et al., 1987; Conger & Conger, 1994; Deater-Deckard et al., 2001), and we expected children to report higher levels of parental differential treatment than parents.

## Method

### Participants

We used data from 632 twin pairs, approximately seven years old (*M* = 7.6, SD = .94, 51.9% female) who participated in the Wisconsin Twin Project (Schmidt et al., 2019). Twin zygosity was 20.3% female MZ, 15.1% male MZ, 16.1% female DZ, 18.2% male DZ, and 30.3% opposite-sex DZ. Zygosity information was not available for 2.8% of the sample. The sample was 96% European American and 1.6% African American; and the remaining participants were Native American, Asian, Pacific Islander, Hispanic, mixed race, or other. 44% of families resided in small towns or villages (<10,000 population) or in rural areas. Mothers and fathers had 14.7 (SD = 4.05) and 14.4 (*SD* = 3.93) years of education, on average, respectively. 33% of fathers and 22% of mothers had only a high school education or less, and 35% of fathers and 39% of mothers had a college degree, with the rest having completed some post-high school training. Financially, in the period of approximately 1996–2008, when twins were initially recruited into the sample, 7.7% of families made less than $30,000 annually, 30.3% made $30,000 to $50,000, 24.6% had an income of $50,000 to $70,000, 27.6% earned from $70,000 to $100,000, and 9.7% earned $100,000 or more per year. According to the U.S. Census Bureau (2024), average household income was $47,122 in 1996 and $68,424 in 2008. The majority of mothers were married (91.7%); but 4.5% were single, and 3.7% were divorced. Fewer fathers than mothers completed reports (*n* = 400 paternal reports; *n* = 546 maternal reports).

### Procedure

Participating families submitted written informed consent and received compensation. Zygosity and demographic information was collected via initial telephone interviews, during which children were also assessed for symptoms of psychopathology by parental report using the MacArthur Health and Behavior Questionnaire (HBQ; Armstrong, Goldstein, & The MacArthur Working Group on Outcome Assessment, 2003). Families also participated in a five-hour home visit in which more data were collected by trained interviewers. Additional parent-reported measures were completed through phone interviews and questionnaire packets, including the Differential Treatment Questionnaire (DTQ; McGuire & Roch-Levecq, 2001) and the Sibling Relationship Questionnaire (SRQ-R; Furman & Buhrmester, 1985).

During the home visits, the twins participated in various activities designed to assess temperament and behavior. An interaction between the primary caregiver (97% mothers, 3% fathers) and the twins was designed to measure parenting. During this interaction, the primary caregiver sat at the kitchen table between her twin children and taught them a matching card game that she had learned from one of the testers. Testers were not present during this interaction. Each child also completed the SRQ puppet interview. These episodes were videotaped for later coding.

### Measures

#### Zygosity

The *Zygosity Questionnaire for Young Twins* (Goldsmith, 1991) contains 32 items and shows 93% to 96% agreement with genotyping (Forget-Dubois et al., 2003). It was used as a parent assessment of the twins’ zygosity during the initial phone interview. Expert ratings based on photos and videos, hospital birth records, and lab placentae reports were also used to confirm zygosity. When zygosity was difficult to determine from other methods, genotyping was used for 2% of families.

#### Differential treatment

PDT was assessed using multiple informants, including mothers, fathers, twins, and trained observers, consistent with evidence that reporter perspectives often diverge (Jeannin & Van Leeuwen, 2015; Meunier et al., 2012). Parents tend to perceive their behavior as more equitable, whereas children – who are especially sensitive to subtle differences – report greater disparity (Atzaba-Poria & Pike, 2008; Kowal et al., 2006; Luo et al., 2020). Both parent- and child-reported PDT are related to children’s psychopathology (Jeannin & Van Leeuwen, 2015; McHale et al., 2000; Meunier et al., 2012; Richmond et al., 2005; Richmond & Stocker, 2008).

*DTQ. The Differential Treatment Questionnaire* (DTQ) was adapted by McGuire and Roch-Levecq (2001) from the MacArthur Longitudinal Twin Study’s interviewing procedures to assess parental differential treatment of infants and toddlers through parent report. Each DTQ subscale contains few items (two for attention and discipline and one for affection), so internal consistency estimates could not be computed. However, prior research has demonstrated moderate temporal stability across approximately year-long periods for affection (*r* = .43), attention (*r* = .32), and discipline (*r* = .33; McGuire & Roch-Levecq, 2001), similar to observational data in infancy and toddlerhood (Dunn et al., 1986). The present data also shows coherent associations across reporters and convergent measures (see Supplemental Tables 4 and 5). For absolute differential treatment, DTQ subscales showed moderate correlations with corresponding measures of parental partiality from the Sibling Relationship Questionnaire (SRQ) and with relevant observational indicators, supporting convergent validity. In contrast, correlations for relative differential treatment were smaller and less consistent across reporters and domains, suggesting weaker convergence for the relative scores. These findings suggest that despite its brevity, the DTQ provides valid indices of differential parental treatment.

The DTQ consists of three sets of questions concerning differential parental attention (2 items), affection (1 item), and discipline (2 items). Questions such as “Do you find that one of them is more difficult to handle than the other?” are answered by the responding parent on a 1–5 scale indicating the direction of which twin is receiving more of a particular kind of treatment (1 = Twin A much more than Twin B; 2 = Twin A a bit more than Twin B; 3 = Both about the same; 4 = Twin B a bit more than Twin A; 5 = Twin B much more than Twin A). Both mothers and fathers completed this questionnaire regarding their own behavior toward the children. Twin A and Twin B were randomly assigned.

These responses were recoded in two ways for this study. *Absolute* scores were used to represent the overall amount of differential treatment exhibited within families, regardless of which twin received the more favorable treatment. Thus, mother and father responses of 1 and 5 were recoded into 3 (much difference), scores of 2 and 4 were recoded into 2 (some difference), and scores of 3 were recoded into 1 (no difference). In addition, *relative* scores were used to represent which child received more or less discipline, attention, or affection on this measure. A score of 3 on the DTQ scales as originally coded indicated that the twins were treated the same, whereas scores higher than 3 indicated more discipline/attention/affection directed towards Twin B, and scores lower than 3 indicated more of that treatment directed towards Twin A. We reverse-coded the items on Twin A’s scales, such that for both twins, higher scores on the *relative* discipline, attention, and affection scales indicated more of the received treatment for oneself, and lower scores indicated more received treatment for one’s cotwin. Relative scores for each twin were dependent on each other, but were used individually to predict psychopathology symptoms after controlling for clustering within families.

*SRQ. The Sibling Relationship Questionnaire* (SRQ; Furman & Buhrmester, 1985) assessed mother and father partiality in parent–child relationships through both mother and child reports. Mother partiality and father partiality are two of the 15 scales on the SRQ, each of which includes three items. For mother reports, the questionnaire’s partiality items were coded on a 1–5 scale (1 = Twin B almost always gets treated better, 3 = The children get treated about the same, 5 = Twin A almost always gets treated better). These scores were recoded into a magnitude 1–3 scale (1 = no partiality, 2 = a bit partiality, 3 = much partiality) as well as a directional 1–5 scale, recoded as described for the DTQ.

Children responded to the same partiality items on the SRQ via a puppet interview format similar to the Berkeley Puppet Interview (BPI; Measelle et al., 1998). These items were later coded on a 1–7 scale by trained, reliable coders (with chance-corrected Kappa agreement greater than .70), ranging from more negative to more positive responses. Thus, an item on the parent SRQ asking “Who gets more attention from mother, Twin A or Twin B?” would correspond with an item on the SRQ puppet interview stating, “My mom gives me more attention than my twin/My mom gives my twin more attention than me.” The child-reported SRQ was also recoded into a 3-point magnitude scale, but since the 1–7 original scale could not be as easily converted into the 3-point scale, the 7-point scale was first divided into five equal segments: extreme scores of 1–2.2 or 5.81–7 were recoded as 3’s (much partiality), more moderate scores of 2.21–3.4 or 4.61–5.8 were recoded as 2’s (some partiality), and scores in the middle ranging from 3.41–4.6 were recoded as 1’s (little or no partiality). The original 1–7 scale was used as the relative score for each twin. In this case, twin scores were not dependent on each other.

Internal consistency estimates (Cronbach’s *α* and mean inter-item correlations) for the 3-item SRQ subscales ranged from .41 to .90 and .20 to .76, respectively. As expected for brief 3-item scales, alphas were low for several mother- and child-reported relative partiality scales (e.g., mother-reported relative maternal partiality, *α* = .47; child-reported relative maternal partiality, *α* = .41), but their mean inter-item correlations were within the acceptable range (*r*s = .20–.35). In contrast, the mother- and child-reported absolute scales showed the strongest internal consistency (e.g., child-reported absolute paternal partiality, *α* = .90, *r* = .76; mother-reported paternal partiality, *α* = .86, *r* = .69). Overall, the pattern of results indicates that while some scales demonstrated lower reliability due to their brevity, item associations were consistent with expectations for the SRQ.

*Observational Measures.* The observational measure of differential treatment was coded from the matching card game episode of the videotaped interactions. The task involved the primary caregiver teaching twins a novel matching card game shown to elicit various parenting behaviors. Separate trained coders with Kappa agreement of greater than .70 recorded the presence or absence of specified parental behaviors within 10-second epochs over a six-minute period of videotape, and 15% of videos were double-coded. Different coders coded each twin. Separate warmth, hostility, and intrusiveness composites were then created for each parent-twin dyad by taking the mean across all 10 second epochs for coded verbal warmth, verbal/nonverbal parental hostility, and verbal/nonverbal intrusiveness, respectively. Parental warmth was coded whenever a parent showed affection or kindness toward the target twin. Parental intrusiveness was coded when parents intervened without the child’s request or restricted the child’s independence. Parental hostility was coded for negative physical interactions, such as grabbing the child’s arm or taking cards out of the child’s hand. In twin models, the original warmth, hostility, and intrusiveness scores were used. For models examining differential treatment, we calculated both relative and absolute differential treatment scores to match the DTQ and SRQ scales. To calculate relative differential treatment for each twin, the cotwin’s score was subtracted from the twin’s score. For example, if Twin A received a 5 on warmth and Twin B received a 3 on warmth, Twin A’s relative score was 2 and Twin B’s relative score was −2. The absolute differential treatment score was created by taking the absolute value of the relative differential treatment score.

#### Symptoms of child psychopathology

Child psychopathology was measured using the MacArthur Health and Behavior Questionnaire (HBQ; Armstrong et al., 2003), an instrument designed to measure psychological symptoms and other functioning in children aged four to eight years. The item content was derived largely from DSM-IV diagnostic criteria. Mothers and fathers responded separately to items regarding each child on a 0–2 scale, indicating “not true for the child” to “very true for the child.” Alphas for the current sample were .90 for internalizing, .92 for externalizing, and .92 for ADHD symptoms. Means of scales were used to form internalizing (depression, over-anxiousness, and separation anxiety; 29 items total), externalizing (conduct disorder, oppositional defiant disorder, and overt aggression; 31 items total), and ADHD (inattention and impulsivity; 15 items total) composites. As mother and father reports of these symptoms were correlated (mean *r* = .55, *p* < .01), mean composites across reporters were formed.

#### Covariates

Age (measured continuously) and sex (measured dichotomously; 1 = female, 2 = male) were included as covariates in regression models. In univariate twin models, age was included as a covariate, and sex-limitation models were estimated to model the effects of sex directly. For bivariate twin models, variables were residualized for age and sex prior to fitting the models. These covariates were chosen because children in middle childhood may be more attuned to PDT (Buist et al., 2013; Steinberg & Monahan, 2007), and twins who are the same sex may expect to be treated similarly, making PDT more salient (Buist et al., 2013; Shanahan et al., 2008). Research suggests that PDT is more strongly related to psychopathology when siblings are closer in age and when both are male (Buist et al., 2013) and to internalizing symptoms when both are female (Shanahan et al., 2008).

### Testing for differences in differential treatment and psychopathology by zygosity

To detect differences in differential treatment or psychopathology symptoms for families with MZ twins, same-sex DZ twins, and opposite-sex DZ twins, one-way ANOVAS were conducted. For measures with individual scores for each twin, cluster robust ANOVAs were conducted to account for clustering within families. There were no differences for any twin-reported measures or for twin psychopathology symptoms by zygosity. There were also no differences for observed measures of parenting. There were differences for maternal reports of absolute maternal discipline, attention, affection, paternal discipline, paternal attention, maternal partiality, and paternal partiality. In all cases, parents reported lower levels of differential treatment for MZ compared to DZ twins (see Supplemental Table 1).

### Aim 1 methodology

Twin intraclass correlations (see Supplemental Table 6) were computed separately for female MZ, female DZ, male MZ, male DZ, and opposite-sex DZ twins for observed parenting because these measures had twin-level variability. There was also twin-level variability for child-reported SRQ, but we elected not to fit twin models to these scales because twin correlations for absolute partiality were near zero and negative, and twin correlations for absolute maternal partiality were small (absolute value of <.15) for all groups except male MZ twins (.28), pointing to primarily nonshared environmental influences. Higher similarity in MZ twins relative to DZ twins is indicative of underlying genetic influences.

The OpenMx package in R (Neale et al., 2003) was used to test a series of nested models representing genetic and environmental influences on scale variation. The primary model estimated values for (A) additive genetic influences, (C) common (shared) environmental influences, and (E) nonshared environmental influences. The full model allowed separate estimates for girls and boys, which were then constrained to be equal in the next model to determine whether or not estimates significantly varied by sex. Qualitative sex-limitation models test whether different genetic or environmental factors influence the trait in males and females (i.e., whether the sources of variance differ by sex). Quantitative sex-limitation models test whether the same genetic and environmental factors operate in both sexes but differ in their magnitude. Finally, no sex-difference models assume that the same genetic and environmental factors influence the trait equally in males and females. Model fit was evaluated using chi-square difference tests, which compared these sex-limitation models and subsequent simplified models that systematically dropped the A and C parameters to assess whether reduced models could be retained without significantly worsening fit. The more parsimonious, simpler models were adopted as the best fitting models when the chi-squared difference tests were nonsignificant.

In addition to univariate ACE models, we conducted bivariate Cholesky decomposition models in OpenMx to examine overlap between genetic and environmental influences on parenting and child psychopathology. Bivariate models were estimated only for observed hostility and ADHD and observed intrusiveness and ADHD, as these pairs of variables were the only ones to show phenotypic correlations greater than .15 (*r* = .17 and *r* = .19) in preliminary analyses. For all models, the individual twin scores for observed parenting and psychopathology were used.

Bivariate Cholesky decompositions estimate the proportion of variance in each trait that is attributable to A, C, and E influences, as well as the extent to which these influences are common across the two traits, allowing an examination of shared etiology between parenting and child characteristics. The model first decomposes the variance in parenting (hostility or intrusiveness) into A, C, and E components (A_1_, C_1_, and E_1_). The variance in ADHD symptoms is then partitioned into variance shared with parenting (via the same A_1_, C_1_, and E_1_ factors) and variance unique to ADHD (through additional A, C, and E factors specific to ADHD, A_2_, C_2_, and E_2_). This structure enables us to identify whether the association between parenting and ADHD symptoms is primarily due to shared genetic or environmental influences. To evaluate model parsimony, we compared fully unconstrained bivariate ACE models with nested models in which specific paths were constrained to be zero. We first dropped all paths that estimated at 0, then systematically tested the remaining shared (A_21_, C_21_, and E_21_) and independent (A_2_, C_2_, and E_2_) paths one at a time, and if any were non-significant, we examined whether both could be dropped together. Best-fitting models were selected based on chi-square difference tests and AIC values. Full information maximum likelihood was used to handle missing data.

### Aim 2 methodology

To account for the interdependence in the data (i.e., twins nested within families), we ran cluster robust standard error regressions, which adjust standard errors to account for clustering in the data. With absolute (Table 5) and relative (Table 6) parental differential treatment, we ran separate models predicting individual twin scores on internalizing, externalizing, and ADHD symptoms. All absolute DTQ scales (Maternal and Paternal Discipline, Attention, and Affection) were included as predictors together in regression models predicting internalizing, externalizing, and ADHD symptoms. The same was true for all absolute SRQ mother- and child-report maternal and paternal partiality scales in a second set of regressions and all observed absolute PDT measures in a third. Regressions predicting child symptoms from the equivalent relative parental differential treatment measures followed the same strategy. Findings for absolute differential treatment reflected family-level associations. All analyses were conducted in the lavaan package in *R* (v0.6-7; Rosseel, 2012), with full information maximum likelihood used to handle missing data.

## Results

### Preliminary analysis: Comparing measures of parental differential treatment

#### Descriptive statistics for absolute and relative differential treatment variables

**Figure 1.**
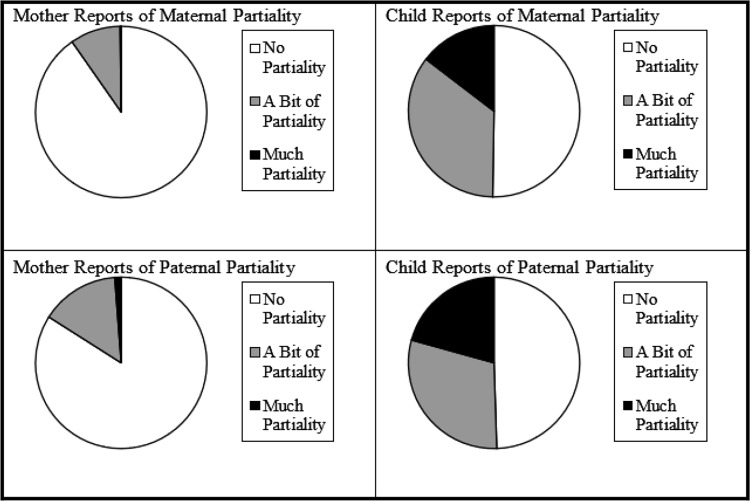
Sibling relationship questionnaire responses. *Note:* No Partiality (composite means from 1–1.66 for twin reports, *M* = 1 for mother reports); A Bit of Partiality (composite means from 1.67–2.33 for twin reports, *M* = 2 for mother reports); Much Partiality (composite means from 2.34–3 for twin reports, *M* = 3 for mother reports).

The means, standard deviations, and ranges are provided in Supplemental Table 2 for family-level absolute and relative measures and Supplemental Table 3 for individual-level variables. Compared to mothers, children reported more SRQ maternal partiality, *t* (428) = −13.71, *p* < .01; *t* (426) = −15.00, *p* < .01, and paternal partiality, *t* (420) = −11.29, *p* < .01; *t* (418) = −12.31, *p* < .01, see Figure 1. Paternal partiality was significantly higher than maternal partiality for both mother-report, *t*(519) = −4.41, *p* < .01, and child-report, *t*(836) = .−3.57, *p* < .01.

#### Correlations across raters and measures

We ran correlations to compare measure agreement and reporter agreement for absolute (Supplemental Table 4) and relative measures (Supplemental Table 5). As hypothesized, mother and father reports of their own differential treatment on the DTQ were significantly correlated across domains, ranging from *r* = .27 (maternal affection and paternal affection) to .54 (maternal discipline and paternal discipline) for absolute ratings and from *r* = –.23 (maternal attention and paternal attention) to .65 (maternal discipline and paternal discipline) for relative ratings (*p*s < .01). Agreement was strongest for discipline and modest for affection, whereas attention showed an inverse association on relative scores. In contrast, mother and child reports of differential treatment were largely independent. Correlations between parent and child reports of parental partiality were negligible across both absolute and relative measures, ranging from *r* = –.07 to .16 (Supplemental Tables 4–5). Although small effects reached significance in a few cases (e.g., paternal partiality, *p* < .01), the overall pattern indicated minimal correspondence between parent and child perceptions of differential treatment.

The correlations between observed parenting measures and the SRQ and DTQ scales were mixed (see Supplemental Tables 4 and 5). Only the absolute DTQ paternal discipline scale was significantly correlated with all three observed measures (observed warmth (*r* = .15), observed intrusiveness (*r* = .15), and observed hostility (*r* = .25). There were mixed findings for observed hostility and intrusiveness. Observed warmth was only correlated with paternal measures.

### Aim 1: Genetic and environmental influences on parental differential treatment

Twin intraclass correlations for observed warmth, hostility, and intrusiveness variables are reported separately by sex in Supplemental Table 6. For warmth (female ICC_MZ_ = .35, male ICC_MZ_ = .57; female ICC_DZ_ = .32, male ICC_DZ_ = .57, opposite sex ICC_DZ_ = .67) and hostility (female ICC_MZ_ = .38, male ICC_MZ_ = .26; female ICC_DZ_ = .36, male ICC_DZ_ = .31, opposite sex ICC_DZ_ = .20), both MZ and DZ cotwins were highly similar suggesting a significant impact of the shared environment, which creates similarities between siblings raised together. For intrusiveness, on the other hand, MZ correlations were higher than DZ correlations for male twins (female ICC_MZ_ = .27, male ICC_MZ_ = .51; female ICC_DZ_ = .32, male ICC_DZ_ = .14, opposite-sex ICC_DZ_ = .35), indicating that this dimension of parenting may be impacted by heritable characteristics of children and suggesting the presence of sex differences.

Findings from biometric models estimating influences on observed parents are listed in Tables 1–3, with the best-fitting, most parsimonious models bolded. For observed parental warmth, the CE model fit best, indicating that both the shared and non-shared environment contributed to parental warmth (Table 1). For intrusiveness, an AE model fit best, indicating that heritable characteristics of children and non-shared environment contributed to observed intrusiveness (Table 2). A sex-limited model fit best for hostility (Table 3), with both A and E contributing to variation in hostility for females, indicating the influence of heritable child characteristics and non-shared environment. For males, C and E contributed to variation in hostility, indicating that the shared and nonshared environments contributed to differences, with little genetic influence. Overall, univariate models showed moderate genetic influence on negative parenting, thus providing evidence that parenting is at least partially child-driven.

**Table 1.** Genetic and environmental influences on observed parental warmth

Model	−2*LL*	*df*	*ΔX* ^2^	*Δdf*	*p*	*AIC*	*Am*	*Cm*	*Em*	*Af*	*Cf*	*Ef*	*rG*
Qualitative sex limitation model	−1,038.64	390	NA	NA	NA	−1,022.64	.01 [0.00, 0.40]	.63 [0.31, 0.75]	.35 [0.22, 0.50]	.00 [0.00, 0.31]	.42 [0.17, 0.57]	.58 [0.42, 0.74]	1.0
Quantitative sex limitation model	−1,038.64	390	0	0	NA	−1,022.64	.01 [0.00, 0.40]	.63 [0.31, 0.75]	.35 [0.22, 0.50]	.00 [0.00, 0.31]	.42 [0.17, 0.57]	.58 [0.42, 0.74]	
							*A*	*C*	*E*				
Full ACE -no sex limitation model	−1,034.02	393	4.62	3	.20	−1,024.02	.00 [0.00, 0.27]	.53 [0.31, 0.63]	.47 [0.36, 0.58]				
Reduced ACE model – no sex limitation model													
CE	−1,034.02	394	< .001	1	1.00	−1,026.02	**–**	.53 [0.43, 0.63]	.47 [0.37, 0.58]				
E	−968.61	395	65.41	2	< .001	−962.61	**–**	**–**	1.00 [1.00, 1.00]				

*Note.* A, C, and E are standardized squared parameter estimates for additive genetic, common environment, and nonshared environmental factors, respectively. The standardized 95% confidence interval is reported in brackets. The most parsimonious model is indicated in bold. 2LL = −2 log likelihood; df = degrees of freedom, *Δ =* change; AIC = Akaike’s information criterion; m = male, f = female; *rG* indicates the correlation between the A factor for male twins and the A factor for female twins.

**Table 2.** Genetic and environmental influences on observed parental intrusiveness

Model	*−*2*LL*	*df*	*ΔX* ^2^	*Δdf*	*p*	*AIC*	*Am*	*Cm*	*Em*	*Af*	*Cf*	*Ef*	*rG*
Qualitative sex limitation model	−1,690.91	405	NA	NA	NA	−1672.92	.41 [0.00, 0.64]	.05 [0.00, 0.49]	.54 [0.36, 0.78]	.25 [0.00, 0.72]	.31 [0.00, 0.67]	.45 [0.27, 0.74]	1.0
Quantitative sex limitation model	−1,690.91	406	<.001	1	1.0	−1674.91	.41 [0.00, 0.64]	.05 [0.00, 0.49]	.54 [0.36, 0.78]	.25 [0.00, 0.72]	.31 [0.00, 0.67]	.45 [0.27, 0.74]	
							*A*	*C*	*E*				
Full ACE – o sex limitation model	−1,683.25	409	7.67	4	.10	−1673.25	.42 [0.00, 0.64]	.08 [0.00, 0.40]	.50 [0.36, 0.69]				
Reduced ACE model- no sex limitation model													
AE	−1,683.03	410	.22	1	.64	−1675.03	.51 [0.36, 0.64]	–	.49 [0.36, 0.64]				
E	−1,651.29	411	31.96	2	< .001	−1645.29	**–**	**–**	1.00 [1.00, 1.00]				

*Note.* A, C, and E are standardized squared parameter estimates for additive genetic, common environment, and nonshared environmental factors, respectively. The standardized 95% confidence interval is reported in brackets. The most parsimonious model is indicated in bold. 2LL = −2 log likelihood; df = degrees of freedom, *Δ* = change; AIC = Akaike’s information criterion; m = male, f = female; *rG* indicates the correlation between the A factor for male twins and the A factor for female twins.

**Table 3.** Genetic and environmental influences on observed parental hostility

Model	*−*2*LL*	*df*	*ΔX* ^2^	*Δdf*	*p*	*AIC*	*Am*	*Cm*	*Em*	*Af*	*Cf*	*Ef*	*rG*
Qualitative sex limitation model	−1,419.53	405	NA	NA	NA	−1,401.53	.06 [0.00, 0.58]	.27 [0.00, 0.51]	.67 [0.42, 0.91]	.34 [0.00, 0.59]	.05 [0.00, 0.46]	.61 [0.40, 0.89]	1.0
Quantitative sex limitation model	−1,419.53	406	< .001	1	1.00	−1,403.53	.06 [0.00, 0.58]	.27 [0.00, 0.51]	.67 [0.42, 0.91]	.34 [0.00, 0.59]	.05 [0.00, 0.46]	.61 [0.40, 0.89]	
							*A*	*C*	*E*				
Full ACE – no sex limitation model	−1,401.22	409	18.31	4	.001	−1,391.22	.29 [0.00, 0.53]	.07 [0.00, 0.36]	.64 [0.47, 0.86]				
Reduced ACE model													
AE	−1,401.10	410	.12	1	.73	−1,393.10	.30 [0.19, 0.53]	**–**	.70 [0.47, 0.81]				
CE	−1,400.00	410	1.17	1	.28	−1,392.05	**–**	.25 [0.12, 0.38]	.75 [0.62, 0.88]				
E	−1,386.31	411	14.91	2	< .001	−1,380.31	**–**	**–**	1.00 [1.00, 1.00]				

*Note.* A, C, and E are standardized squared parameter estimates for additive genetic, common environment, and nonshared environmental factors, respectively. The standardized 95% confidence interval is reported in brackets. The most parsimonious model is indicated in bold. 2LL = −2 log likelihood; df = degrees of freedom, *Δ* = change; AIC = Akaike’s information criterion; m = male, f = female; *rG* indicates the correlation between the A factor for male twins and the A factor for female twins.

Next, we used bivariate twin models to investigate whether the heritable influences on parenting are captured, at least in part, by child psychopathology symptoms. All bivariate models were combined across sex to conserve statistical power. Fit statistics for the full models and final reduced models are reported in Table 4. These models revealed that the covariation between observed intrusiveness and ADHD and observed hostility and ADHD was predominately explained by additive genetics. In the full model, the additive genetic factors for intrusiveness and ADHD were correlated at .43. For hostility, they were correlated at .17. These findings suggest that genetic differences in children elicit parenting through a process of evocative rGE.

**Table 4.** Shared genetic influences between observed intrusive and hostile parenting and children’s ADHD

Intrusiveness with ADHD															
	*−*2*LL*	*df*	*AIC*	*Δ−2LL*	*Δ df*	*p*	*A1*	*A2*	*C1*	*C2*	*E1*	*E2*	*rA*	*rC*	rE
ACE	−603.51	1617	−3,837.21	–	–	–	.37		.13		.50		.43	−1.0	-.04
ACE–ACE							.13	.59	.01	.00	.00	.27			
ACE	−603.16	1620	−3,843.16	.34	3	.95	.39		.11		.50		.35	–	–
A–AE							.09	.64	–	–	–	.27			
AE	−602.74	1621	−3,844.74	.77	4	.94	.52		–		.48		.30	–	–
A–AE							.07	.66	–	–	–	.27			
AE	−574.00	1622	−3,818.00	29.50	5	< .001									
A–E															
Hostility with ADHD															
	*−2LL*	*df*	*AIC*	*Δ−2LL*	*Δ df*	*p*	*A1*	*A2*	*C1*	*C2*	*E1*	*E2*	*rA*	*rC*	*rE*
ACE	−306.47	1617	−3,540.47	–	–	–	.36		.02		.62		.17	1.0	.09
ACE–ACE							.02	.71	.00	.00	.00	.27			
AE	−305.62	1621	−3,547.62	.86	4	.93	.39		–		.61		.23	–	–
A-AE							.04	.69	–	–	–	.27			
AE	−291.07	1622	−3,535.07	15.40	5	.01									
A–E															

*Note.* The model column indicates which parameters are estimated in the model. The first set of ACE parameters represent A, C, and E influences on phenotype 1, the second set represents influences on Phenotype 1 that are shared with Phenotype 2, and the third set represent A, C, and E influences on Phenotype 2 not shared with Phenotype 1. Thus, ACE-ACE-ACE indicates the full model with all parameters estimated, whereas ACE-A-AE indicates a model dropping shared C and E, and independent C. Phenotype 2 is ADHD. −2LL = −2 log likelihood; Δ = change; AIC = Akaike’s Information Criterion; A1, C1, and E1 are additive genetic variance, shared environmental variance, and nonshared environmental variance in variable 1, which may be shared with variable 2; A2, C2, and E2 = represent variance in variable 2 that is independent of variable 1; rA, rC, and rE represent the correlations between the latent A (or C, or E) factors influencing each phenotype; The 1.00 and −1.00 shared environmental correlations in the full models are due to these correlations being calculated using near −0 estimates of C variance in phenotype 2. Unstandardized variance components are reported, followed by standardized variance components. *The variance was calculated from a negative path estimate.

### Aim 2: Parental differential treatment and psychopathology symptoms

#### Absolute parental differential treatment (Table 5)

Our second goal was to relate reports of parental differential treatment to child symptoms of internalizing, externalizing, and ADHD. Absolute differences in maternal discipline positively predicted internalizing, externalizing, and ADHD symptoms, whereas absolute differences in paternal discipline only positively predicted externalizing symptoms. Absolute differences in paternal partiality rated by mothers positively predicted internalizing and ADHD symptoms, and absolute differences in paternal affection were related to internalizing symptoms. Finally, absolute differences in observed intrusiveness positively predicted ADHD symptoms.

**Table 5.** Absolute differential treatment as a predictor of symptoms of child psychopathology – cluster robust standard error corrected regressions

	HBQ internalizing			HBQ externalizing			HBQ ADHD		
	*B [CI]*	*SE*	*t*	*B[CI]*	*SE*	*t*	*B[CI]*	*SE*	*t*
DTQ maternal discipline	.09*** [.05, .12]	.02	4.44	.10*** [.06, .14]	.02	4.84	.17*** [.10, .24]	.04	4.80
DTQ maternal attention	.07 [.00, .13]	.03	1.99	.03 [−.05, .10]	.04	.72	.06 [−.04, .16]	.05	1.13
DTQ maternal affection	−.01 [−.04, .02]	.01	−.75	.00 [−.03, .04]	.02	.10	−.02 [−.07, .03]	.02	−.92
DTQ paternal discipline	.03 [−.01, .07]	.02	1.47	.05* [.001, .10]	.02	2.11	.05 [−.02, .13]	.04	1.46
DTQ paternal attention	.04 [−.01, .01]	.02	1.60	.01 [−.04, .06]	.03	.37	−.00 [−.08, .07]	.04	−.12
DTQ paternal affection	.03* [.00, .06]	.01	2.04	.01 [−.02, .04]	.02	.59	.02 [−.03, .07]	.03	.73
SRQ maternal partiality - M	.05 [−.02, .12]	.04	1.31	.08 [−.01, .17]	.05	1.77	.05 [−.06, .16]	.06	.93
SRQ paternal partiality - M	.10** [.03, .14]	.03	2.81	.05 [−.00, .11]	.03	1.88	.10* [.02, .19]	.04	2.35
SRQ maternal partiality -C	−.00 [−.03, .03]	.01	−.17	−.01 [−.04, .02]	.02	−.65	-.03 [−.09, .02]	.03	−1.25
SRQ paternal partiality - C	.02 [−.01, .05]	.01	1.29	.03 [−.00, .05]	.01	1.81	.03 [−.01, .08]	.02	1.44
Warmth observed	.07 [−.58, .72]	.33	.21	−.11 [−.82, .59]	.36	−.32	.42 [−.62, 1.46]	.53	.79
Intrusiveness observed	.08 [−1.03, 1.19]	.56	.14	.49 [−.81–1.79]	.66	.74	2.85* [.52, 5.17]	1.18	2.40
Hostility observed	.57 [−.13, 1.26]	.35	1.61	.64 [−.23, 1.52]	.45	1.44	1.06 [−.10, 2.23]	.60	1.79

*Note.* HBQ = MacArthur Health and Behavior Questionnaire; DTQ = Differential Treatment Questionnaire (1 = no difference, 2 = some difference, 3 = much difference); SRQ = Sibling Relationship Questionnaire (1 = no partiality, 2 = a bit of partiality, 3 = much partiality); M = mother report, C = child report.; *CI* = 95% confidence interval; *p < .05; **p < .01; *** *p* < .001. Age and gender were included as controls in all regressions. Regressions are separated by borders.

#### Relative parental differential treatment (Table 6)

Relative DTQ maternal discipline also positively predicted internalizing, externalizing, and ADHD symptoms, suggesting that the child who receives more discipline compared to their twin had higher psychopathology symptoms. Similar to the absolute measures, relative paternal discipline also positively predicted externalizing symptoms, but it additionally predicted ADHD symptoms. Lastly, relative maternal partiality reported by mothers positively predicted externalizing symptoms, meaning that the twin favored by mothers had higher externalizing symptoms.

**Table 6.** Relative differential treatment as a predictor of symptoms of child psychopathology – cluster robust standard error corrected regressions

	HBQ internalizing			HBQ externalizing				HBQ ADHD	
	*B*	*SE*	*t*	*B*	*SE*	*t*	*B*	*SE*	*t*
DTQ maternal discipline	.03*** [.02, .05]	.01	4.26	.04*** [.03, .06]	.01	6.58	.07*** [.05, .09]	.01	5.89
DTQ maternal attention	.04 [−.00, .09]	.02	1.93	.02 [−.01, .05]	.02	1.31	.02 [−.05, .08]	.03	.55
DTQ maternal affection	−.00 [−.02, .01]	.01	−.31	−.01 [−.02, .01]	.01	−1.07	.00 [−.02, .03]	.01	.30
DTQ paternal discipline	.00 [−.02, .02]	.01	.17	.05*** [.03, .06]	.01	6.97	.07*** [.05, .10]	.01	5.12
DTQ paternal attention	−.01 [−.04, .02]	.02	−.47	.01 [−.01, 03]	.01	1.06	.00 [−.04, .04]	.02	.11
DTQ paternal affection	.01 [−.00, .02]	.01	1.71	−.01 [−.02, .00]	.01	−1.56	.01 [−.01, .03]	.01	.74
SRQ maternal partiality-M	−.02 [−.07, .04]	.03	−.59	.06* [.00, .11]	.03	2.09	.01 [−.08, .10]	.05	.14
SRQ paternal partiality-M	.04 [−.00, .08]	.02	1.94	−.00 [−.04, .03]	.02	−.14	−.03 [−.10, .03]	.03	−.98
SRQ maternal partiality-C	.01 [−.01, .02]	.01	.66	−.01 [−.03, .01]	.01	−.83	−.01 [−.04, .02]	.01	−.65
SRQ paternal partiality-C	−.00 [−.02, .02]	.01	−.28	.00 [−.02, .02]	.01	.07	.01 [−.02, .03]	.01	.54
Warmth observed	−.00 [−.27, .27]	.14	−.03	.21 [−.03, .46]	.13	1.69	.24 [−.14, .62]	.19	1.24
Intrusiveness observed	.51 [−.04, 1.07]	.29	1.81	.33 [−.19, .85]	.26	1.26	.53 [−.44, 1.49]	.49	1.07
Hostility observed	−.29 [−.66, .08]	.19	−1.55	.09 [−.37, .54]	.23	.38	.11 [−54, .76]	.33	.32

*Note.* HBQ = MacArthur Health and Behavior Questionnaire; DTQ = Differential Treatment Questionnaire; SRQ = Sibling Relationship Questionnaire; M = mother report, C = child report.; *p < .05; *CI* = 95% confidence interval; **p < .01; *** *p* < .001. Age and gender were included as controls in all regressions. Regressions are separated by borders.

In general, both the absolute and relative measures of parental differential treatment were associated with symptoms of child psychopathology, with differences in maternal discipline and differences in paternal affection and partiality standing out as salient. Children’s perceptions of maternal and paternal partiality were not related to psychopathology symptoms.

## Discussion

Based on univariate twin models, observed warmth was primarily explained by the shared environment and non-shared environment; observed hostility estimates varied with sex and were explained by additive genetics, shared environment, and non-shared environment; and observed intrusiveness was explained by additive genetics and non-shared environment. Bivariate twin models showed shared genetic estimates for observed hostility and ADHD symptoms, as well as intrusiveness and ADHD symptoms. Overall, our findings supported the hypotheses that parental differential treatment is more strongly related to externalizing symptoms than internalizing symptoms and that differences in observed negative parenting are more associated with psychopathology symptoms than differences in positive parenting (Boyle et al., 2004; Jenkins et al., 2003; Meunier et al., 2012; Richmond & Stocker, 2008; Richmond et al., 2005).

### Preliminary analysis: Reporter and measurement agreement

When comparing reporters and observational assessment of differential treatment, we hypothesized that children would report higher levels of parental differential treatment than parents and the measures of parental differential treatment from the DTQ and the SRQ would be correlated. Our hypotheses were confirmed. Children reported significantly higher partiality than mothers, and DTQ and SRQ measurements were consistent. Children may report higher partiality than mothers because they are more attuned to subtle differences in parenting (Kowal et al., 2006). Part of this could be due to the developmental period being studied, as children in middle childhood tend to think concretely about the world and may not fully understand the nuances of why they are being treated differently (Piaget, 1954).

Parents’ reports on the DTQ and SRQ were also correlated, indicating consistency in how parents identify a favored child. However, this finding should be cautiously interpreted because measures were completed within the same questionnaire packet. This limitation does not apply to child reports of maternal and paternal partiality, which were completed independently. Further, we found associations between the SRQ and DTQ scales and observed parenting measures. Absolute levels of paternal discipline were correlated with all three absolute observed measures, perhaps suggesting that paternal discipline is a good indicator of visible parenting behaviors. Interestingly, the only other significant correlations for observed warmth were also with paternal measures on the SRQ and DTQ scales. This suggests that paternal differential treatment is a particularly effective metric for detecting variability in observed positive parenting.

Based on our findings on reporter and measurement agreement, we recommend that future studies use either the SRQ or DTQ, supplemented with observational measures when possible. We also recommend utilizing maternal and paternal report of differential treatment for future studies aimed to detect differences in psychopathology. Although child reports of differential treatment showed higher levels of differential treatment, only maternal and paternal reports predicted psychopathology. Child reports of differential treatment may reflect momentary assessments of parental fairness (Kowal et al., 2006), but they may be less accurate in predicting ongoing parenting patterns that could contribute to psychopathology.

### Aim 1: Genetic and environmental contributions to the association

Biometric twin modeling helped us better understand the genetic and environmental factors influencing parenting. Variations in observed hostility towards males were less heritable and showed a significant influence from shared environmental factors. Differences in observed hostility towards females and differences in observed intrusiveness for males and females were mainly explained by additive genetic factors, indicating that children’s heritable traits were eliciting specific parenting behaviors. Shared environment accounted for differences in observed parental warmth. Warmth was not related to symptoms of child psychopathology. Overall, based on both child- and observer-reported parenting data, parenting is influenced by both environmental factors and genetics.

Bivariate twin models built on these findings by assessing the shared A, C, and E estimates between observed parenting and ADHD symptoms. The models showed that the covariance between observed hostility and ADHD, as well as observed intrusiveness and ADHD, were mainly explained by shared genetics. In other words, the genetics linked to higher ADHD symptoms were also connected to higher parental hostility and intrusiveness. This type of evocative gene–environment correlation suggests that children are eliciting certain parenting behaviors rather than parenting causing the child’s psychopathology. While some parents may react negatively to their children’s heritable ADHD symptoms, others may not. In fact, interventions that focus on parenting ADHD children explicitly focus on reducing negative parenting that is elicited by children’s ADHD symptoms (Friedman et al., 2020).

### Aim 2: Parent differential treatment and child psychopathology symptoms

Our second goal was to examine how reports of parental differential treatment relate to child symptoms of internalizing, externalizing, and ADHD. We hypothesized that both absolute and relative differences in parental discipline, hostility, and intrusiveness would predict externalizing and ADHD symptoms and, to a lesser extent, internalizing symptoms. We also predicted that overall maternal and paternal partiality would predict higher internalizing, externalizing, and ADHD symptoms. Our hypotheses were only partially supported.

#### Maternal discipline

Consistent with our hypotheses, absolute differences in maternal discipline were associated with internalizing, externalizing, and ADHD symptoms. When twins receive different discipline from their mother, there may be more tension and inconsistency in the home overall. This aligns with the current literature which has found that disorganized or inconsistent parenting is associated with internalizing, externalizing, and ADHD symptoms (Badovinac et al., 2021). We also found support for social comparison theory as relative differences in maternal discipline were associated with internalizing, externalizing, and ADHD symptoms for the sibling who is less favored. These findings could be child-driven where the child with higher psychopathology symptoms elicits higher discipline. However, past longitudinal research controlling for earlier symptoms of psychopathology has found that the association between parental differential treatment and child psychopathology symptoms was driven by differential treatment (Richmond et al., 2005; Richmond & Stocker, 2008; Shanahan et al., 2008). Taken together, these findings support differential maternal discipline as an important factor to consider when studying psychopathology.

#### Paternal discipline

We also expected that absolute and relative differences in paternal discipline would be associated with psychopathology symptoms, and this expectation was mostly but not entirely supported. Absolute differences in paternal discipline were associated with externalizing symptoms, and relative differences in paternal discipline were associated with externalizing symptoms and ADHD symptoms, but neither measure was associated with internalizing symptoms. Paternal discipline may be associated with externalizing symptoms but not internalizing symptoms because paternal parenting is generally perceived as more rule-based, authoritarian, restrictive, and harsh (Yaffe et al., 2023). Children may respond to this harsher treatment with visible acts of rebellion. For example, if one sibling is punished and another is not, the punished sibling may intentionally break the rules to protest the differences in discipline. Internalizing symptoms may be more likely to develop in response to daily differences in discipline. Mothers typically spend more time with their children and handle everyday discipline matters (Hofferth et al., 2012), which could explain why maternal discipline, but not paternal discipline, is associated with internalizing symptoms. The findings could also reflect child-driven parenting, where a child acting out more prompts increased discipline. The results for paternal discipline support our hypothesis that parental differential treatment would be more strongly associated with externalizing symptoms than internalizing ones.

#### Maternal and paternal partiality

Based on current literature, we expected that absolute, but not relative, maternal and paternal partiality would predict internalizing, externalizing, and ADHD symptoms. In our study, higher absolute paternal partiality, rated by mothers, predicted higher internalizing symptoms and ADHD symptoms, while relative maternal partiality, also rated by mothers, predicted higher externalizing behavior for the more favored child. This is counter to our hypothesis that absolute favoritism from both mothers and fathers would be associated with these symptoms. There is evidence that fathers exert a unique influence on their children compared to mothers (Jeynes, 2016), so this might be an instance where paternal influence plays a more significant role. Considering past research that shows mixed findings on favoritism (Jeannin & Van Leeuwen, 2015; Luo et al., 2020; Meunier et al., 2012), these results should be interpreted cautiously. For relative favoritism, we initially expected no significant associations, but we found a notable one: the mother’s favored child had higher externalizing symptoms. The favored child may show higher externalizing behaviors because favoritism can increase their sense of entitlement, which could lead to acting out if they do not get their way and treating others with less respect (Finzi-Dottan & Cohen, 2010). Overall, the main takeaway is that both relative and absolute measures of favoritism can be related to psychopathology and that being the favored child does not always correspond to lower levels of these symptoms.

#### Observed intrusiveness and hostility

For observed measures of parenting, we partially replicated the significant associations between intrusiveness and hostility and psychopathology found in the literature (Eisenberg et al., 2015; Gluschkoff et al., 2017). Supporting Aim 2, absolute differences in observed intrusiveness predicted ADHD symptoms, but absolute differences in observed hostility did not predict psychopathology symptoms. Although only absolute levels of observed intrusiveness were related to ADHD, these findings have important implications. A recent meta-analysis on parenting children with ADHD showed that higher ADHD symptom severity in children was significantly linked to increased parental intrusiveness (Claussen et al., 2024). The association between absolute intrusiveness and ADHD may capture family-level characteristics, such as parental stress, emotional exhaustion, or overall inconsistency, which can contribute both to elevated ADHD symptoms and greater differential treatment. Another explanation is that biological parents of children with ADHD may exhibit higher levels of ADHD-related traits such as impulsivity which contribute to higher levels of intrusiveness (Friedman et al., 2020). It is also possible that we only observed significant associations with ADHD symptoms because there is more variation in ADHD symptoms at age seven compared to internalizing and externalizing symptoms.

### Limitations and future directions

This study provides a unique examination of parental differential treatment by analyzing parenting from both parent-driven and child-driven perspectives, supported by objective coding of a videotaped card game interaction. Additionally, it includes multiple reporters and measures of parental differential treatment and is robust in representing rural families. However, caution is advised when generalizing these findings to other populations due to a lack of diversity in race and ethnicity, as well as the fact that the siblings are twins. Parenting behaviors may be applicable to non-twin samples where siblings are close in age (Mönkediek et al., 2020), but they might not be relevant for families with siblings who are farther apart in age. Moreover, families living in poverty are underrepresented.

Methodologically, the SRQ paternal partiality report was completed by mothers, and there is limited understanding of the reliability of the DTQ measure. Additionally, while the bivariate models reflected results from the full sample, prior sex-limitation models indicate that sex differences in parental hostility may exist and require further investigation. Finally, high levels of observed intrusive and hostile parenting were rare, and in both cases, the small group of twins at the upper extreme likely influenced the results. Replication of the observed parenting findings with a larger sample would better represent children experiencing high levels of negative parenting compared to their peers and increase confidence in the findings.

In future studies, researchers should include observational measures and parental favoritism assessments, as there is limited information on these methods. Additionally, research indicates that perceptions of fairness in treatment are important moderators (Kowal et al., 2006) and could have practical implications for improving child outcomes. If parents can help their children understand why they are being treated differently, it may reduce the risk of psychopathology symptoms. Future studies should also examine different family structures to better understand differences between maternal and paternal differential treatment. In addition, other genetically informed designs, such as children-of-twins studies, could help clarify whether associations between parent behaviors and child outcomes reflect environmental influences or shared genetic factors. Lastly, although most of the literature focuses on the relationship between parental differential treatment and psychopathology from a parent-driven perspective, there is evidence that child-driven effects are also significant. Genetically informed research, such as twin modeling, will be crucial to understanding these effects.

## Supporting information

10.1017/S0954579426101370.sm001 Maravilla et al. supplementary material Maravilla et al. supplementary material

## Data Availability

De-identified data are available from the study principal investigators upon reasonable request. All relevant code for this study’s analyses is available at https://osf.io/gew9r.

## Supplementary material

The supplementary material for this article can be found at https://doi.org/10.1017/S0954579426101370.

## Preregistration

Analyses were not preregistered as data analysis began before preregistration was standard practice.

## References

[ref2] Ansbacher, H. L. , & Ansbacher, R. R. (1956). The individual psychology of Alfred Adler. Basic Books, Inc.

[ref3] Armstrong, J. M. , Goldstein, L. H. , & The MacArthur Working Group on Outcome Assessment. (2003). Manual for the MacArthur Health and Behavior Questionnaire (HBQ 1.0). University of Pittsburgh. MacArthur Foundation Research Network on Psychopathology and Development (David J. Kupfer, Chair).

[ref4] Atzaba-Poria, N. , & Pike, A. (2008). Correlates of parental differential treatment: Parental and contextual factors during middle childhood. Child Development, 79(1), 217–232. 10.1111/j.1467-8624.2007.01121.x 18269519

[ref5] Avinun, R. , & Knafo, A. (2014). Parenting as a reaction evoked by children’s genotype: A meta-analysis of children-as-twins studies. Personality & Social Psychology Review, 18(1), 87–102. 10.1177/10888683134983 23940232

[ref6] Badovinac, S. D. , Pillai Riddell, R. , Deneault, A. A. , Martin, J. , Bureau, J. F. , & O’Neill, M. C. (2021). Associations between early childhood parent–child attachment and internalizing/externalizing symptoms: A systematic review and narrative synthesis. Marriage & Family Review, 57(7), 573–620. 10.1080/01494929.2021.1879984

[ref7] Bell, R. Q. (1968). A reinterpretation of the direction of effects in studies of socialization. Psychological Review, 75(2), 81. 10.1037/h0025583 4870552

[ref8] Boyle, M. H. , Jenkins, J. M. , Georgiades, K. , Cairney, J. , Duku, E. , & Racine, Y. (2004). Differential-maternal parenting behavior: Estimating within-and between-family effects on children. Child Development, 75(5), 1457–1476. 10.1111/j.1467-8624.2004.00751.x 15369525

[ref9] Brody, G. H. , Stoneman, Z. , & Burke, M. (1987). Child temperaments, maternal differential behavior, and sibling relationships. Developmental Psychology, 23(3), 354–362. 10.1037/0012-1649.23.3.354

[ref11] Bronfenbrenner, U. , & Ceci, S. J. (1994). Nature-nurture reconceptualized in developmental perspective: A bioecological model. Psychological Review, 101(4), 568. 10.1037/0033-295X.101.4.568 7984707

[ref12] Buist, K. L. , Deković, M. , & Prinzie, P. (2013). Sibling relationship quality and psychopathology of children and adolescents: A meta-analysis. Clinical Psychology Review, 33(1), 97–106. 10.1016/j.cpr.2012.10.007 23159327

[ref13] Carbonneau, R. , Eaves, L. J. , Silberg, J. L. , Simonoff, E. , & Rutter, M. (2002a). Assessment of the within-family environment in twins: Absolute versus differential ratings, and relationship with conduct problems. Journal of Child Psychology & Psychiatry, 43(8), 1064–1074. https://doi-org.ezproxy1.lib.asu.edu/10.1111/1469-7610.00233 12455927 10.1111/1469-7610.00233

[ref14] Carbonneau, R. , Rutter, M. , Silberg, J. L. , Simonoff, E. , & Eaves, L. J. (2002b). Assessment of genetic and environmental influences on differential ratings of within-family experiences and relationships with twins. Psychological Medicine, 32, 729–741. 10.1017/S0033291702005639 12102387

[ref15] Claussen, A. H. , Holbrook, J. R. , Hutchins, H. J. , Robinson, L. R. , Bloomfield, J. , Meng, L. , & Kaminski, J. W. (2024). All in the family? A systematic review and meta-analysis of parenting and family environment as risk factors for attention-deficit/hyperactivity disorder (ADHD) in children. Prevention Science, 25(Suppl 2), 249–271. 10.1007/s11121-022-01358-4 35438451 PMC9017071

[ref16] Conger, K. J. , & Conger, R. D. (1994). Differential parenting and change in sibling differences in delinquency. Journal of Family Psychology, 8(3), 237–302. 10.1037/0893-3200.8.3.287

[ref17] Daniels, D. , & Plomin, R. (1985). Differential experience of siblings in the same family. Developmental Psychology, 5, 747–760. 10.1037/0012-1649.21.5.747

[ref18] Deater-Deckard, K. , Pike, A. , Petrill, S. A. , Cutting, A. L. , Hughes, C. , & O’Connor, T. G. (2001). Nonshared environmental processes in social-emotional development: An observational study of identical twin differences in the preschool period. Developmental Science, 4(2), F1–F6. 10.1111/1467-7687.00157

[ref68] Dunn, J. F. , Plomin, R. , & Daniels, D. (1986). Consistency and change in mothers behavior toward young siblings. Child Development, 57, 348–356. 10.2307/1130590 3956317

[ref20] Eisenberg, N. , Taylor, Z. E. , Widaman, K. F. , & Spinrad, T. L. (2015). Externalizing symptoms, effortful control, and intrusive parenting: A test of bidirectional longitudinal relations during early childhood. Development and Psychopathology, 4(1), 953–968. 10.1017/S0954579415000620 26439056

[ref21] Eradus, M. , Leijten, P. , Melendez-Torres, G. J. , Foo, X. Q. , & Oliver, B. R. (2024). Parental differential warmth, hostility, and sibling differences in internalizing and externalizing behavior problems: A meta-analysis. Journal of Family Psychology, 38(3), 387–399. 10.1037/fam0001194 38271066

[ref23] Festinger, L. (1954). A theory of social comparison processes. Human Relations, 7(2), 117–140. 10.1177/001872675400700202

[ref24] Finzi-Dottan, R. , & Cohen, O. (2010). Young adult sibling relations: The effects of perceived parental favoritism and narcissism. The Journal of Psychology, 145(1), 1–22. 10.1080/00223980.2010.528073 21290927

[ref25] Forget-Dubois, N. , Pérusse, D. , Turecki, G. , Girard, A. , Billette, J.-M. , Rouleau, G. , Boivin, M. , Malo, J. , & Tremblay, R. E. (2003). Diagnosing zygosity in infant twins: Physical similarity, genotyping, and chorionicity. Twin Research, 6(6), 479–485. 10.1375/136905203322686464 14965457

[ref26] Friedman, L. M. , Dvorsky, M. R. , McBurnett, K. , & Pfiffner, L. J. (2020). Do parents’ ADHD symptoms affect treatment for their children? The impact of parental ADHD on adherence to behavioral parent training for childhood ADHD. Journal of Abnormal Child Psychology, 48(11), 1425–1437. 10.1007/s10802-020-00672-1 32813210 PMC7567125

[ref27] Furman, W. , & Buhrmester, D. (1985). Children’s perceptions of the personal relationships in their social networks. Developmental Psychology, 21, 1016–1024. 10.1037/0012-1649.21.6.1016

[ref28] Gluschkoff, K. , Keltikangas-Järvinen, L. , Pulkki-Råback, L. , Jokela, M. , Viikari, J. , Raitakari, O. , & Hintsanen, M. (2017). Hostile parenting, parental psychopathology, and depressive symptoms in the offspring: A. 32-year follow-up in the Young Finns study. Journal of Affective Disorders, 208, 436–442. 10.1016/j.jad.2016.11.002 27855296

[ref29] Goldsmith, H. H. (1991). A zygosity questionnaire for young twins: A research note. Behavior Genetics, 21, 257–269. 10.1007/BF01065819 1863259

[ref31] Hofferth, S. L. , Pleck, J. , Stueve, J. L. , Bianchi, S. , & Sayer, L. (2012). The demography of fathers: What fathers do. In N. J. Cabrera , & C. S. Tamis-LeMonda (Eds.), Handbook of Father Involvement (pp. 79–106). Routledge.

[ref32] Jeannin, R. , & Van Leeuwen, K. (2015). Associations between direct and indirect perceptions of parental differential treatment and child socio-emotional adaptation. Journal of Child & Family Studies, 24(6), 1838–1855. 10.1007/s10826-014-9987-3

[ref33] Jenkins, J. M. , Rasbash, J. , & O’Connor, T. G. (2003). The role of the shared family context in differential parenting. Developmental Psychology, 39(1), 99–113. 10.1037/0012-1649.39.1.99 12518812

[ref34] Jensen, A. C. , & Thomsen, A. E. (2024). Parental differential treatment of siblings linked with internalizing and externalizing behavior: A meta-analysis. Child Development, 95, 1384–1405. https://doi-org.ezproxy1.lib.asu.edu/10.1111/cdev.14091 38439142 10.1111/cdev.14091

[ref35] Jeynes, W. H. (2016). Meta-analysis on the roles of fathers in parenting: Are they unique? Marriage & Family Review, 52(7), 665–688. 10.1080/01494929.2016.1157121

[ref36] Kessler, R. C. , Berglund, P. , Demler, O. , Jin, R. , Merikangas, K. R. , & Walters, E. E. (2005). Lifetime prevalence and age-of-onset distributions of DSM-IV disorders in the National Comorbidity Survey Replication. Archives of General Psychiatry, 62(6), 593–602. 10.1001/archpsyc.62.6.593 15939837

[ref37] Kowal, A. , Kramer, L. , Krull, J. L. , & Crick, N. R. (2002). Children’s perceptions of the fairness of parental preferential treatment and their socioemotional well-being. Journal of Family Psychology, 16(3), 297. 10.1037/0893-3200.16.3.297 12238412

[ref38] Kowal, A. K. , Krull, J. L. , & Kramer, L. (2006). Shared understanding of parental differential treatment in families. Social Development, 15(2), 276–295. 10.1046/j.1467-9507.2006.00341.x

[ref39] Lemery, K. S. , & Goldsmith, H. H. (1999). Genetically informative designs for the study of behavioural development. International Journal of Behavioral Development, 23, 293–317. 10.1080/01650259938383

[ref40] Loehlin, J. C. (1996). The Cholesky approach: A cautionary note. Behavior Genetics, 26, 65–69. 10.1007/BF02361160

[ref41] Loehlin, J. C. , Neiderhiser, J. M. , & Reiss, D. (2005). Genetic and environmental components of adolescent adjustment and parental behavior: A multivariate analysis. Child Development, 76(5), 1104–1115. 10.1111/j.1467-8624.2005.00900.x 16150005

[ref42] Luo, R. , Chen, F. , Yuan, C. , Ma, X. , & Zhang, C. (2020). Parent–child discrepancies in perceived parental favoritism: Associations with children’s internalizing and externalizing problems in Chinese families. Journal of Youth and Adolescence, 49(1), 60–73. 10.1007/s10964-019-01148-2 31889229

[ref43] McGuire, S. , & Roch-Levecq, A. C. (2001). Mothers’ perceptions of differential treatment of infant twins. In R. M. Emde , & J. K. Hewitt (Eds.), Infancy to Early Childhood (pp. 247–256). New York, New York: Oxford University Press.

[ref44] McHale, S. M. , Updegraff, K. A. , Jackson-Newsom, J. , Tucker, C. J. , & Crouter, A. C. (2000). When does parents’ differential treatment have negative implications for siblings? Social Development, 9(2), 149–172. 10.1080/0165025993838

[ref45] Measelle, J. R. , Ablow, J. C. , Cowan, P. A. , & Cowan, C. P. (1998). Assessing young children’s views of their academic, social, and emotional lives: An evaluation of the self-perception scales of the Berkeley Puppet Interview. Child Development, 69, 1556–1576. 10.1111/j.1467-8624.1998.tb06177.x 9914640

[ref46] Meunier, J. C. , Roskam, I. , Stievenart, M. , Van De Moortele, G. , Browne, D. T. , & Wade, M. (2012). Parental differential treatment, child’s externalizing behavior and sibling relationships: Bridging links with child’s perception of favoritism and personality, and parents’ self-efficacy. Journal of Social & Personal Relationships, 29(5), 612–638. 10.1177/0265407512443419

[ref47] Mönkediek, B. , Schulz, W. , Eichhorn, H. , & Diewald, M. (2020). Is there something special about twin families? A comparison of parenting styles in twin and non-twin families. Social Science Research, 90, 102441. 10.1016/j.ssresearch.2020.102441 32825925

[ref69] Neale, M. C., Boker, S. M., Xie, G., & Maes, H. H. (2003). Mx: Statistical Modeling (6th edn.). Department of Psychiatry, Virginia Commonwealth University.

[ref48] Papachristou, E. , & Flouri, E. (2020). The codevelopment of internalizing symptoms, externalizing symptoms, and cognitive ability across childhood and adolescence. Development and Psychopathology, 32(4), 1375–1389. 10.1017/S0954579419001330 31588887

[ref49] Piaget, J. (1954). The development of object concept. In J. Piaget , & M. Cook (Trans.) (Eds.), The Construction of Reality in the Child (pp. 3–96). Basic Books.

[ref50] Pike, A. , Manke, B. , Reiss, D. , & Plomin, R. (2000). A genetic analysis of differential experiences of adolescent siblings across three years. Social Development, 9(1), 96–114. 10.1111/1467-9507.00113

[ref51] Plomin, R. , & Daniels, D. (1987). Why are children in the same family so different from one another? Behavioral and Brain Sciences, 10(1), 1–16. 10.1017/S0140525X00055941

[ref52] Plomin, R. , DeFries, J. C. , & Loehlin, J. C. (1977). Genotype-environment interaction and correlation in the analysis of human behavior. Psychological Bulletin, 84(2), 309. https://doi-org.ezproxy1.lib.asu.edu/10.1037/0033-2909.84.2.309 557211

[ref54] Richmond, M. K. , & Stocker, C. M. (2008). Longitudinal associations between parents’ hostility and siblings’ externalizing behavior in the context of marital discord. Journal of Family Psychology, 22(2), 231. 10.1037/0893-3200.22.2.231 18410210

[ref53] Richmond, M. K. , Stocker, C. M. , & Rienks, S. L. (2005). Longitudinal associations between sibling relationship quality, parental differential treatment, and children’s adjustment. Journal of Family Psychology, 19(4), 550. 10.1037/0893-3200.19.4.550 16402870

[ref55] Rietveld, M. J. , Hudziak, J. J. , Bartels, M. , Van Beijsterveldt, C. E. M. , & Boomsma, D. I. (2003). Heritability of attention problems in children: I. Cross-sectional results from a study of twins, age 3-12 years. American Journal of Medical Genetics Part B: Neuropsychiatric Genetics, 117(1), 102–113. 10.1002/ajmg.b.10024 12555244

[ref56] Rosseel, Y. (2012). Lavaan: An R package for structural equation modeling. Journal of Statistical Software, 48(2), 1–36. http://www.jstatsoft.org/v48/i02/

[ref57] Schachar, R. , & Tannock, R. (1995). Test of four hypotheses for the comorbidity of attention-deficit hyperactivity disorder and conduct disorder. Journal of the American Academy of Child & Adolescent Psychiatry, 34(5), 639–648. 10.1097/00004583-199505000-00016 7775359

[ref58] Schmidt, N. L. , Lemery-Chalfant, K. , & Goldsmith, H. H. (2019). Wisconsin twin project overview: Temperament and affective neuroscience. Twin Research and Human Genetics, 22(6), 794–799. 31818344 10.1017/thg.2019.108 PMC7056557

[ref59] Shanahan, L. , McHale, S. M. , Crouter, A. C. , & Osgood, D. W. (2008). Linkages between parents’ differential treatment, youth depressive symptoms, and sibling relationships. Journal of Marriage and Family, 70(2), 480–494. 10.1111/j.1741-3737.2008.00495.x

[ref60] Steinberg, L. , & Monahan, K. C. (2007). Age differences in resistance to peer influence. Developmental Psychology, 43(6), 1531. 10.1037/0012-1649.43.6.1531 18020830 PMC2779518

[ref61] Substance Abuse and Mental Health Services Administration (SAMHSA). (2021). Key substance use and mental health indicators in the United States: Results from the 2020 National Survey on Drug Use and Health (HHS Publication No. PEP21-07-01-003, NSDUH Series H-56). Rockville, MD: Center for Behavioral Health Statistics and Quality, Substance Abuse and Mental Health Services Administration. https://www.samhsa.gov/data/

[ref62] Towers, H. , Spotts, E. , Neiderhiser, J. M. , Plomin, R. , Mavis Hetherington, E. , & Reiss, D. (2000). Genetic and environmental influences on teacher ratings of the Child Behavior Checklist. International Journal of Behavioral Development, 24(3), 373–381.

[ref63] Turkheimer, E. , & Waldron, M. (2000). Nonshared environment: A theoretical, methodological, and quantitative review. Psychological Bulletin, 126(1), 78. 10.1037/0033-2909.126.1.78 10668351

[ref64] U.S. Census Bureau. (2024). Table A-2. Mean household income received by each fifth and top 5 percent, 1967 to 2023 [Data set]. U.S. Department of Commerce. https://www.census.gov/data/tables/time-series/demo/income-poverty/historical-income-households.html

[ref66] Whitney, D. G. , & Peterson, M. D. (2019). US national and state-level prevalence of mental health disorders and disparities of mental health care use in children. Journal of the American Medical Association Pediatrics, 173(4), 389–391. 10.1001/jamapediatrics.2018.5399 30742204 PMC6450272

[ref67] Yaffe, Y. (2023). Systematic review of the differences between mothers and fathers in parenting styles and practices. Current Psychology, 42(19), 16011–16024. 10.1007/s12144-020-01014-6

