# Supplementary material for: Parental differential treatment and symptoms of child psychopathology: A twin study
Source: Dev Psychopathol. 2026 May 5:1–15. Online ahead of print. doi: 10.1017/S0954579426101370 (PMC13222760; doi:10.1017/S0954579426101370)
Supplement: Maravilla et al. supplementary material [file S0954579426101370sup001.docx]

Supplemental Materials

# **Supplemental Table 1** *ANOVA Results - Comparing Parental Differential Treatment in MZ, Same Sex DZ, and Opposite Sex DZ Families*

| Measure | *Mean* (*SD*) | | | *F*(*df*) | *p* | η² |
| --- | --- | --- | --- | --- | --- | --- |
|  | MZ | SS - DZ | OS - DZ |  | | |
| Internalizing Symptoms | .30 (.23) | .34 (.25) | .33 (.25) | 1.89 (607) | .153 | .005 |
| Externalizing Symptoms | .28 (.26) | .32 (.27) | .30 (.28) | 1.80 (607) | .166 | .004 |
| ADHD Symptoms | .55 (.42) | .61 (.42) | .58 (.44) | 1.30(607) | .274 | .003 |
| A - Maternal Discipline | 1.49 (.46)_ab_ | 1.76 (.52)_a_ | 1.89 (.60)_b_ | 27.44 (534) | < .001 | .09 |
| A - Maternal Attention | 1.07 (.19)_ac_ | 1.19 (.35)_ac_ | 1.29 (.39)_bc_ | 21.06 (534) | < .001 | .07 |
| A - Maternal Affection | 1.36 (.56)_a_ | 1.72 (.78)_ab_ | 1.39 (.63)_b_ | 16.37 (534) | < .001 | .06 |
| A - Paternal Discipline | 1.42 (.43)_ab_ | 1.63 (.52)_a_ | 1.75 (.54)_b_ | 14.17 (391) | < .001 | .07 |
| A - Paternal Attention | 1.15 (.31)_ab_ | 1.26 (.38)_ac_ | 1.43 (.49)_bc_ | 16.41 (391) | < .001 | .08 |
| A - Paternal Affection | 1.48 (.60) | 1.59 (.66) | 1.41 (.63) | 2.63 (391) | .073 | .01 |
| A- Maternal Partiality (M) | 1.06 (.18)_ab_ | 1.13 (.27)_a_ | 1.14 (.30)_b_ | 4.77 (520) | .009 | .02 |
| A - Paternal Partiality (M) | 1.11 (.29)_a_ | 1.19 (.40) | 1.25 (.43)_a_ | 6.08 (512) | .002 | .02 |
| A - Maternal Partiality (C) | 1.59 (.72) | 1.67 (.74) | 1.67 (.70) | 1.06 (454) | .346 | .003 |
| A - Paternal Partiality (C) | 1.64 (.77) | 1.74 (.81) | 1.77 (.78) | 1.83 (443) | .161 | .005 |
| R - Maternal Partiality (C) | 4.25 (.87) | 4.18 (.93) | 4.18 (.90) | 0.57 (454) | .57 | .001 |
| R – Paternal Partiality (C) | 4.24 (.93) | 4.20 (1.04) | 4.25 (1.03) | 0.23 (443) | .799 | .001 |
| Warmth | .05 (.05) | .06 (.04) | .05 (.04) | 0.59 (196) | .555 | .01 |
| Intrusiveness | .02 (.02) | .03 (.02) | .03 (.02) | 1.91 (204) | .151 | .02 |
| Hostility | .02 (.02) | .03 (.02) | .03 (.03) | 1.91 (204) | .151 | .002 |

*Note.* A = Absolute, C = Child Report, M = Mother Report, MZ = Monozygotic twins. SS-DZ = Same-sex Dizygotic twins. OS-DZ = Opposite-sex Dizygotic twins, df = within-group degrees of freedom. Different superscripts (e.g., a, b) indicate significantly different means based on Tukey’s HSD post hoc test (p < .05) or Games-Howell post hoc test (p <.05), when equal variances were not assumed. Tukey tests were used for Absolute Paternal Attention and Absolute Maternal Partiality (mother report). Games-Howell tests were used for all other significant comparisons. If the subscripts are matching, then there are significant differences between those groups.

**Supplemental Table 2**

*Means and Standard Deviations for Study Variables- Family Level Variables*

|  | Mean (SD) | Range | N |
| --- | --- | --- | --- |
| DTQ Maternal Discipline -Absolute | 1.7 (.55) | 1 – 3 | 546 |
| DTQ Maternal Attention- Absolute | 1.18 (.33) | 1 – 3 | 546 |
| DTQ Maternal Affection – Absolute | 1.49 (.68) | 1 – 3 | 546 |
| DTQ Paternal Discipline -Absolute | 1.58 (.51) | 1 – 3 | 400 |
| DTQ Paternal Attention – Absolute | 1.27 (.40) | 1 – 3 | 400 |
| DTQ Paternal Affection – Absolute | 1.51 (.64) | 1 – 3 | 400 |
| SRQ Maternal Partiality-M – Absolute | 1.11 (.26) | 1 – 3 | 529 |
| SRQ Paternal Partiality-M – Absolute | 1.18 (.38) | 1 – 3 | 520 |
| Observed Differences in Warmth -Absolute | .05 (.05) | 0 - 0.20 | 199 |
| Observed Differences in Intrusiveness -Absolute | .03 (.02) | 0 - 0.12 | 207 |
| Observed Differences in Hostility -Absolute | .04 (.04) | 0 - 0.27 | 207 |
| DTQ Maternal Discipline -Relative | 2.99 (.96) | 1 – 5 | 546 |
| DTQ Maternal Attention- Relative | 2.97 (.33) | 1.5 – 4 | 546 |
| DTQ Maternal Affection – Relative | 2.98 (.84) | 1 – 5 | 546 |
| DTQ Paternal Discipline -Relative | 3.06 (.85) | 1 – 5 | 400 |
| DTQ Paternal Attention – Relative | 3.02 (.47) | 1 – 5 | 400 |
| DTQ Paternal Affection – Relative | 2.98 (.81) | 1 – 5 | 400 |
| SRQ Maternal Partiality-M – Relative | 3.02 (.26) | 1 – 4.33 | 529 |
| SRQ Paternal Partiality-M – Relative | 3.01 (.40) | 1.33 – 5 | 520 |

*Note.* DTQ = Differential Treatment Questionnaire; SRQ = Sibling Relationship Questionnaire; HBQ = MacArthur Health and Behavior Questionnaire; M = mother report

**Supplemental Table 3**

*Means and Standard Deviations for Study Variables – Individual Level Variables*

|  | *Mean (SD)* Sample 1 | *Range* Sample 1 | *N*  Sample 1 | *Mean (SD)* Sample 2 | *Range* Sample 2 | *N*  Sample 2 |
| --- | --- | --- | --- | --- | --- | --- |
| SRQ Maternal Partiality-C – Relative | 4.22 (.88) | 2 -6.33 | 434 | 4.19 (.93) | 2 – 6.33 | 434 |
| SRQ Paternal Partiality-C -Relative | 4.27 (.96) | 2 – 6.33 | 419 | 4.19 (1.03) | 2 – 6.67 | 420 |
| SRQ Maternal Partiality-C – Absolute | 1.62 (.72) | 1 – 3 | 434 | 1.67 (.72) | 1 – 3 | 434 |
| SRQ Paternal Partiality-C -Absolute | 1.68 (.77) | 1 – 3 | 419 | 1.74 (.80) | 1 – 3 | 420 |
| HBQ Internalizing | .31 (.24) | 0 – 1.52 | 621 | .33 (.24) | 0 -1.43 | 620 |
| HBQ Externalizing | .30 (.27) | 0 – 1.64 | 621 | .30 (.27) | 0 – 1.72 | 621 |
| HBQ ADHD | .57 (.42) | 0 – 1.93 | 621 | .59 (.44) | 0 – 2 | 621 |

*Note.* DTQ = Differential Treatment Questionnaire; SRQ = Sibling Relationship Questionnaire; HBQ = MacArthur Health and Behavior Questionnaire; M = mother report, C = child report.

**Supplemental Table 4**

*Reporter and Measure Agreement on Absolute Differential Treatment -Correlations*

|  | 1. | 2. | 3. | 4. | 5. | 6. | 7. | 8. | 9. | 10. | 11. | 12. | 13. | 14. | 15. | 16. |
| --- | --- | --- | --- | --- | --- | --- | --- | --- | --- | --- | --- | --- | --- | --- | --- | --- |
| 1. DTQ Maternal Discipline | 1.00 | .19^**^ | .13^**^ | .54^**^ | .14^**^ | .04 | .29^**^ | .25^**^ | .09^*^ | .08^*^ | .04 | .14^**^ | .16^**^ | .26^**^ | .28^**^ | .27^**^ |
| 2. DTQ Maternal Attention |  | 1.00 | .11^**^ | .13^**^ | .28^**^ | -.00 | .23^**^ | .17^**^ | -.00 | .03 | -.05 | -.00 | .08 | .14^**^ | .08^**^ | .10^**^ |
| 3. DTQ Maternal Affection |  |  | 1.00 | .10^**^ | .00 | .27^**^ | .13^**^ | .12^**^ | .01 | .02 | .02 | .01 | .01 | .06 | .10^**^ | .06 |
| 4. DTQ Paternal Discipline |  |  |  | 1.00 | .18^**^ | .08^*^ | .12^**^ | .17^**^ | .03 | .08^*^ | .15* | .15^**^ | .25^**^ | .21^**^ | .25^**^ | .21^**^ |
| 5. DTQ Paternal Attention |  |  |  |  | 1.00 | .09^**^ | .10^**^ | .22^**^ | .06 | .09 | -.04 | .02 | .03 | .14^**^ | .10 | .04 |
| 6. DTQ Paternal Affection |  |  |  |  |  | 1.00 | .02 | .04 | .11^**^ | .08^*^ | .18^**^ | -.17^**^ | .08 | .10^**^ | .06 | .05 |
| 7. SRQ Maternal Partiality- M |  |  |  |  |  |  | 1.00 | .46^**^ | .01 | .05 | .07 | -.07 | .06 | .10^**^ | .15^**^ | .10^**^ |
| 8. SRQ Paternal Partiality- M |  |  |  |  |  |  |  | 1.00 | .07^*^ | .16^**^ | .07 | -.04 | .11^*^ | .20^**^ | .22^**^ | .20^**^ |
| 9. SRQ Maternal Partiality - C |  |  |  |  |  |  |  |  | 1.00 | .69^**^ | -.08 | .04 | .09 | .05 | .06 | .02 |
| 10. SRQ Paternal Partiality – C |  |  |  |  |  |  |  |  |  | 1.00 | -.04 | -.04 | .08 | .08^*^ | .10^**^ | .06 |
| 11. Observed Warmth |  |  |  |  |  |  |  |  |  |  | 1.00 | -.09 | .09 | .01 | -.03 | .02 |
| 12. Observed Intrusiveness |  |  |  |  |  |  |  |  |  |  |  | 1.00 | .15^**^ | .02 | .07 | .19^**^ |
| 13. Observed Hostility |  |  |  |  |  |  |  |  |  |  |  |  | 1.00 | .10^*^ | .11^*^ | .17^**^ |
| 14. Internalizing HBQ |  |  |  |  |  |  |  |  |  |  |  |  |  | 1.00 | .49^***^ | .52^***^ |
| 15. Externalizing HBQ |  |  |  |  |  |  |  |  |  |  |  |  |  |  | 1.00 | .70^***^ |
| 16. ADHD HBQ |  |  |  |  |  |  |  |  |  |  |  |  |  |  |  | 1.00 |

*Note.* SRQ = Sibling Relationship Questionnaire; DTQ = Differential Treatment Questionnaire; M = mother report, C = child report; **p* < .05; ***p* < .01

**Supplemental Table 5**

*Reporter and Measure Agreement on Relative Differential Treatment – Correlations*

|  | 1. | 2. | 3. | 4. | 5. | 6. | 7. | 8. | 9. | 10. | 11. | 12. | 13. | 14. | 15. | 16. |
| --- | --- | --- | --- | --- | --- | --- | --- | --- | --- | --- | --- | --- | --- | --- | --- | --- |
| 1. DTQ Maternal Discipline | 1.00 | -.02 | -.18^**^ | .65^**^ | .02 | -.11^**^ | .02 | .07^*^ | -.06 | .05 | .09 | .20^**^ | .05 | .13^**^ | .30^**^ | .29^**^ |
| 2. DTQ Maternal Attention |  | 1.00 | .17^**^ | -.02 | -.23^**^ | .09^**^ | -.17^**^ | .03 | .07^*^ | -.07^*^ | .05 | .01 | -.07 | .05 | -.03 | .01 |
| 3. DTQ Maternal Affection |  |  | 1.00 | -.19^**^ | .00 | .37^**^ | -.20^**^ | .01 | .05 | -.04 | .04 | -.01 | -.01 | -.01 | -.10^**^ | -.04 |
| 4. DTQ Paternal Discipline |  |  |  | 1.00 | -.05 | -.18^**^ | -.04 | .10^**^ | .02 | .07 | .12^*^ | .07 | 0.10 | .10^**^ | .31^**^ | .28^**^ |
| 5. DTQ Paternal Attention |  |  |  |  | 1.00 | .10^**^ | .18^**^ | -.23^**^ | -.07 | .18^**^ | -.02 | -.05 | .09 | -.02 | .03 | .04 |
| 6. DTQ Paternal Affection |  |  |  |  |  | 1.00 | .04 | -.08^*^ | -.07 | .02 | .04 | -.01 | -.16** | .03 | -.09^*^ | -.03 |
| 7. SRQ Maternal Partiality- M |  |  |  |  |  |  | 1.00 | -.25^**^ | -.04 | .02 | .07 | -.13^**^ | -.08 | -.02 | .04 | -.00 |
| 8. SRQ Paternal Partiality- M |  |  |  |  |  |  |  | 1.00 | -.02 | -.07^*^ | -.17^**^ | .08 | -.08 | .06^*^ | -.00 | -.01 |
| 9. SRQ Maternal Partiality - C |  |  |  |  |  |  |  |  | 1.00 | .15^**^ | -.04 | -.08 | -.04 | .04 | -.02 | -.01 |
| 10. SRQ Paternal Partiality – C |  |  |  |  |  |  |  |  |  | 1.00 | .06 | -.05 | .04 | -.02 | .01 | .02 |
| 11. Observed Warmth |  |  |  |  |  |  |  |  |  |  | 1.00 | .06 | .21^**^ | -.01 | .07 | .06 |
| 12. Observed Intrusiveness |  |  |  |  |  |  |  |  |  |  |  | 1.00 | .16 | -.02 | .04 | .07 |
| 13. Observed Hostility |  |  |  |  |  |  |  |  |  |  |  |  | 1.00 | .08 | .06 | .08 |
| 14. Internalizing HBQ |  |  |  |  |  |  |  |  |  |  |  |  |  | 1.00 | .49^***^ | .52^***^ |
| 15.Externalizing HBQ |  |  |  |  |  |  |  |  |  |  |  |  |  |  | 1.00 | .70^***^ |
| 16. ADHD HBQ |  |  |  |  |  |  |  |  |  |  |  |  |  |  |  | 1.00 |

*Note.* SRQ = Sibling Relationship Questionnaire; DTQ = Differential Treatment Questionnaire; M = mother report, C = child report; **p* < .05; ***p* < .01

**Supplemental Table 6**

*Twin Intraclass Correlations for Observed Parenting*

|  | Female MZ | Female DZ | Male MZ | Male DZ | OS DZ |
| --- | --- | --- | --- | --- | --- |
| Warmth-Observed | .35 | .32 | .57 | .59 | .67 |
| Intrusiveness-Observed | .27 | .32 | .51 | .14 | .35 |
| Hostility-Observed | .38 | .36 | .26 | .31 | .20 |

*Note.* MZ = monozygotic, DZ = dizygotic, OS = opposite sex twins. SRQ = Sibling Relationship Questionnaire; HBQ = MacArthur Health and Behavior Questionnaire
